# “Sharing the matrix” – a cooperative strategy for survival in *Salmonella enterica* serovar Typhimurium

**DOI:** 10.1186/s12866-023-02972-0

**Published:** 2023-08-23

**Authors:** Kavi Bharathi R., Srinandan C. S., Sai Subramanian N

**Affiliations:** 1 Biofilm Biology Lab, Centre for Research in Infectious Diseases, School of Chemical and Biotechnology, SASTRA Deemed to be University, Thanjavur, Tamil Nadu India; 2 Antimicrobial Resistance Lab, Centre for Research in Infectious Diseases, School of Chemical and Biotechnology, SASTRA Deemed to Be University, Tamil Nadu, Thanjavur, Tamil Nadu 613401 India

**Keywords:** Biofilm, *Salmonella* typhimurium, Matrix, Antimicrobial stress, Cellulose, Curli

## Abstract

**Background:**

Bacteria in nature live together in communities called biofilms, where they produce a matrix that protects them from hostile environments. The components of this matrix vary among species, with *Salmonella enterica* serovar Typhimurium (STm- WT) primarily producing curli and cellulose, which are regulated by the master regulator *csgD*. Interactions between bacteria can be competitive or cooperative, with cooperation more commonly observed among the kin population. This study refers to STm- WT as the generalist which produces all the matrix components and knockout strains that are defective in either curli or cellulose as the specialists, which produces one of the matrix components but not both. We have asked whether two different specialists will cooperate and share matrix components during biofilm formation to match the ability of the generalist which produces both components.

**Results:**

In this study, the response of the specialists and generalist to physical, chemical, and biological stress during biofilm formation is also studied to assess their abilities to cooperate and produce biofilms like the generalist. STm WT colony biofilm which produces both the major biofilm matrix component were protected from stress whereas the non-matrix producer (*∆csgD)*, the cellulose, and curli alone producers *∆csgA,* *∆bcsA* respectively were affected. During the exposure to various stresses, the majority of killing occurred in *∆csgD.* Whereas the co-culture (*∆csgA: ∆bcsA)* was able to resist stress like that of the STm WT. Phenotypic and morphological characteristics of the colonies were typed using congo red assay and the Influence of matrix on the architecture of biofilms was confirmed by scanning electron microscopy.

**Conclusion:**

Our results show that matrix aids in survival during antibiotic, chlorine, and predatory stress. And possible sharing of the matrix is occurring in co-culture, with one counterbalancing the inability of the other when confronted with stress.

**Supplementary Information:**

The online version contains supplementary material available at 10.1186/s12866-023-02972-0.

## Introduction

*Salmonella enterica* is a major causative agent of bacterial food-borne diseases, and it can form biofilms on a variety of biotic and abiotic surfaces [1]. Bacteria adopt a biofilm lifestyle in their natural habitats, where they metamorphose from single planktonic cells to biofilm communities [2, 3]. Biofilm is a sessile mode of life where the microbes aggregate together and live in proximity in a protective self-produced matrix. Biofilm is the predominant life form on Earth. Biofilms protect the bacteria from external stress factors like antibiotics, antibacterial agents, and the immune system of the host [2].

In developing countries, more than 300,000 deaths occur annually due to *Salmonella* infections [4]. Salmonellosis related foodborne illness has been estimated to cost 4.8 to 23 billion dollars annually in the United States [5]. Based on the surface antigenic composition *Salmonella enterica* is classified into 2000 different serovars of which, *Salmonella enterica* serovar Typhimurium (STm), causes non-typhoidal, self-limiting gastroenteritis in humans [1, 6]. Most of these serotypes can adapt to the host including humans. The lifecycle of *Salmonella* revolves between the multicellular sessile communities (biofilm) and the motile planktonic form. This transition aids in the survival, pathogenesis, and transmission of the bacteria from environment to the host, as the biofilm lifestyle protects *Salmonella* from diverse assaults. Population variation is seen between the cells of *Salmonella* which are present inside the host where the planktonic one is used for motility and virulence, whereas the biofilm type for long term survival. *Salmonella* biofilms pose a great nuisance to the food and water distribution systems, because of the resilience conferred by the matrix to chlorine and other water sanitising agents. In health care settings, *Salmonella* biofilms posits life threatening food borne illness and biofilm formation in gall stones [7].

**Fig. 1:**
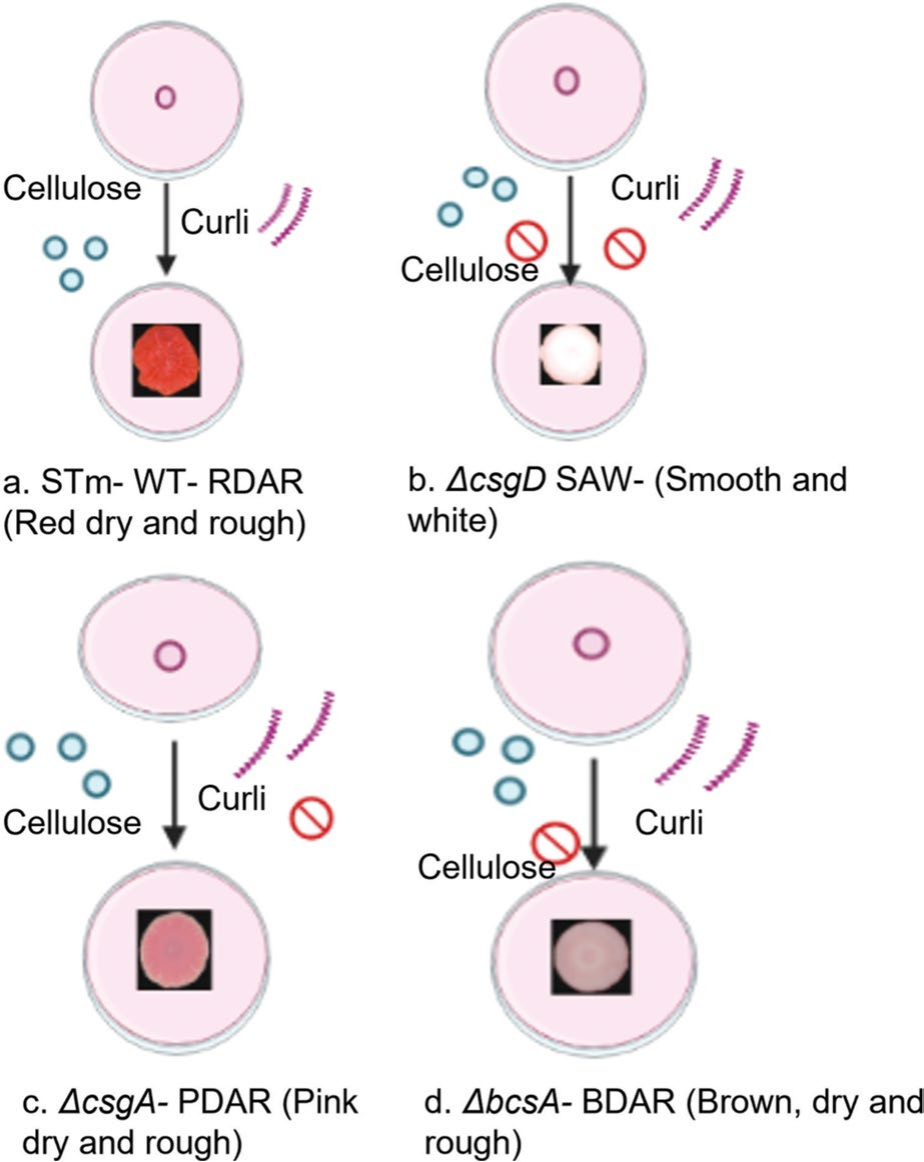
Morphotypes displayed on Congo red agar. **a** STm spotted on Congo red media produce RDAR (Red dry and rough phenotype), **b** *∆csgD* produces SAW (Smooth and white phenotype), **c ***∆csgA* produces PDAR (Pink dry and rough phenotype), d. *∆bcsA* produces BDAR (Brown dry and rough phenotype)

The matrix components play a vital role in the architecture of biofilms [3]. The *Salmonella enterica* serovar Typhimurium (STm) biofilm matrix consists of amyloid protein curli and polysaccharide cellulose, extracellular DNA, and a larger protein *BapA* [8]. The STm wild type (WT) biofilm produces red dry and rough (rdar) morphotype on congo red media (Fig. 1), due to the production of all the matrix components (cellulose, curli). The *csgD*, the master regulator of matrix components positively regulates the production of curli, and cellulose which are the main matrix components of STm biofilm. It is responsible for the virulence of bacteria and plays an important part in biofilm related infections. *csgD* activates *csgBAC* which leads to the production of curli subunits. The expression of *csgD* is in turn regulated by various stimuli like cyclic di-GMP, temperature, etc. High levels of cyclic di GMP promote biofilm formation and vice versa [6].

It has been observed that microbes act collectively in groups for their own benefit and survival. Interactions like competition and cooperation have been observed among them to carry out different tasks. The biofilm matrix where the cells are aggregated have plenty of opportunities for resource sharing, which is a favourable interaction [9]. *∆csgA* is a curli knockout specialist that exclusively produces cellulose alone and *∆bcsA* is a cellulose knockout strain that in turn is specialised to produce curli alone (Fig. 1).

In the present study, we have evaluated whether mutants lacking each one of the matrix components when co-cultured together exhibits sharing of matrix components and do the co-cultured mutants resemble the wild type (which produces both the matrix components) in terms of its resilience to both biotic and abiotic stressors.

## Results

### Frequency of the subtypes

#### Pellicle biofilm, submerged, and colony biofilm

The ability of the STm WT strain to contribute to heterogeneous variants when grown in the form of pellicle, submerged and colony biofilms was estimated by congo red assay. The relative proportion of variants viz., RDAR (Red dry and rough) phenotype, SAW (Smooth and white), and PDAR (Pink dry and rough) cells were estimated for each type of the biofilm. All 3 biofilms were predominated by RDAR/SAW phenotype, but their relative proportions varied within each biofilm (Fig. S1). It was observed that in pellicle biofilms, more SAW phenotype was observed relative to RDAR (Red dry and rough) phenotype. In submerged biofilms RDAR phenotype was predominant but SAW (Smooth and white) and PDAR (Pink dry and rough) cells were also observed. Colony biofilms displayed primarily RDAR Phenotypes followed by SAW (Smooth and white cells), colony biofilms were also comprised of Red big colonies and hyper swarmers. Thus, it appears that the matrix composition varies depending upon the type of biofilm formed, with pellicle biofilm producing relatively less amount of cellulose and curli, which is enhanced in submerged and colony biofilms as evidenced by the preponderance of RDAR phenotype in submerged and colony biofilms.

### Crystal violet assay

**Fig. 2:**
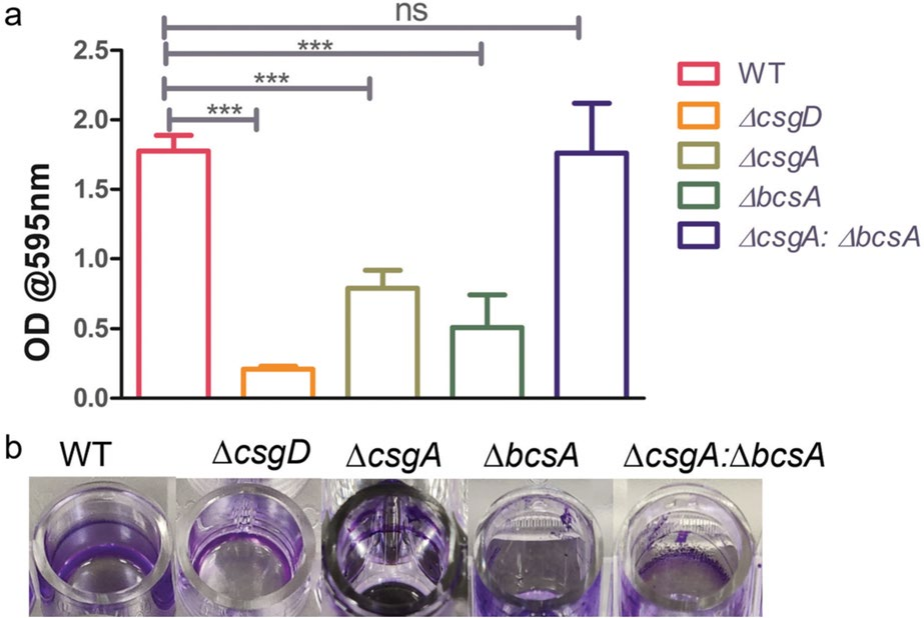
**a** Biofilm forming ability of WT and matrix mutants discerned by Crystal Violet assay. *n* = 3, Unpaired *t-*test was performed. 95% CI, *P* value < 0.0001. **b** the wells showing the crystal violet staining of the biofilm produced

The biofilms formed by the different matrix deficient strains and their co-culture relative to wild type were estimated using crystal violet assay. The observations show that *∆csgD* lacking both the matrix components does not form biofilm. *∆csgA* producing only cellulose and *∆bcsA* producing only curli formed weak biofilms, Importantly the co-culture was able to produce biofilm biomass similar to the wild type (Fig. 2).

### Morphology changes devoid of matrix

**Fig. 3:**
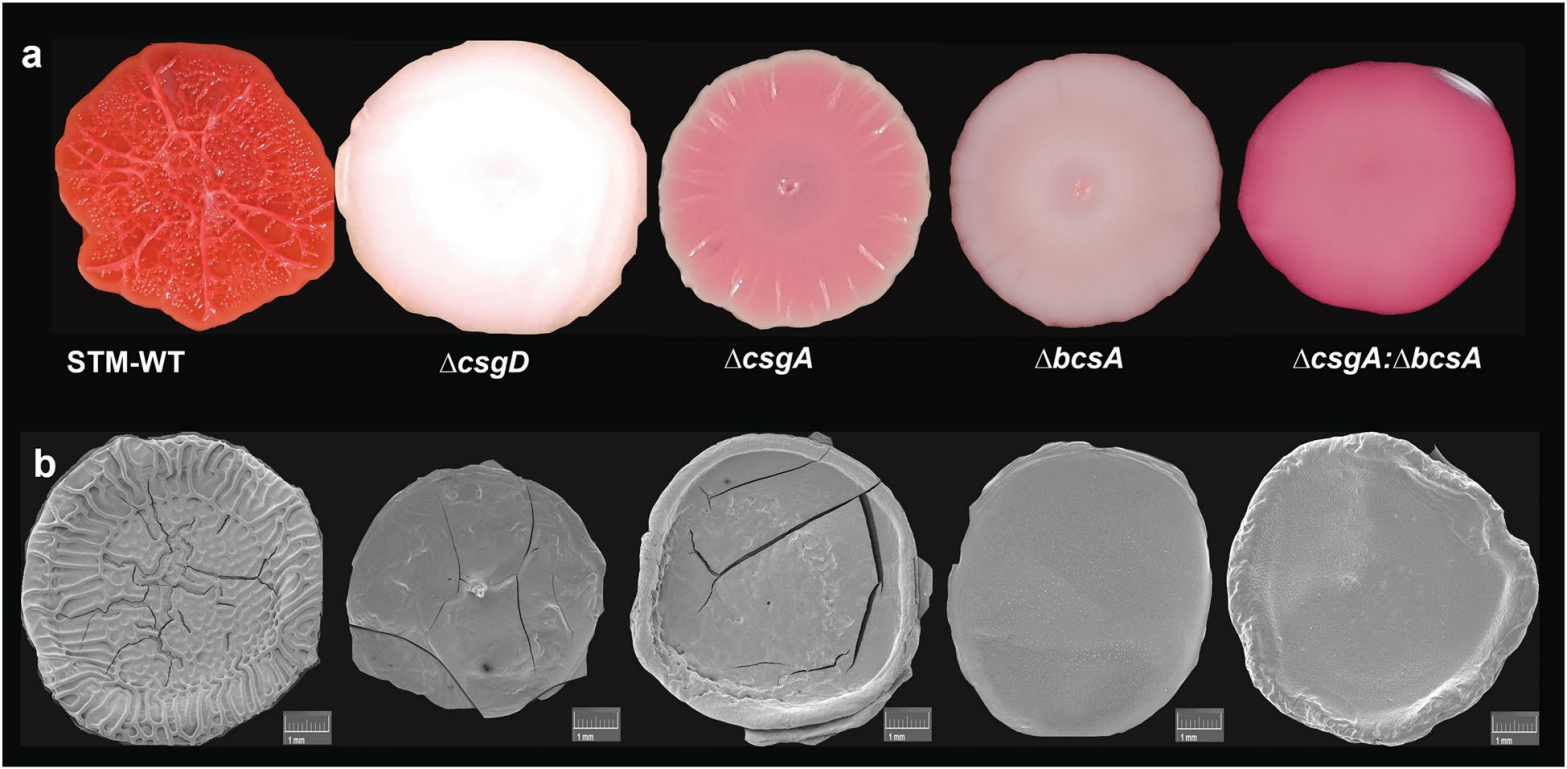
**a** Phenotype of wild type & mutants on Congo Red Media. The 3-day old colony biofilm of STm WT, *∆csgD, ∆csgA, ∆bcsA*, co-culture of *∆csgA: ∆bcsA* spotted on congo red media. **b** Scanning electron microscopic images depicting the morphology of wild type, mutants & co-culture- (Scale bar: 1 mm)

Colony biofilms of STm, WT, and matrix mutants were formed on congo red agar plates, and morphotypes formed were observed. In line with the previous reports, STm WT produces RDAR Red dry and rough morphotype on congo red agar plates where both the matrix components curli and cellulose are expressed, *∆csgD* produces SAW smooth and white phenotype where both curli and cellulose are absent, *∆csgA* produces PDAR (Pink dry and rough) morphotype where only cellulose is present (Fig. 3a). Interestingly signs of septum like (wrinkly) structures which is well developed in the wild type is observed only in *∆csgA* mutant implying that cellulose predominantly contributes to the mature biofilm architecture. *∆bcsA produces* BDAR (Brown dry and rough) morphotype where only curli is present. The co-culture of matrix mutants *∆csgA: ∆bcsA* produces pink colonies where possible sharing of the matrix could have occurred. We added cellulose exogenously to *∆bcsA to* check whether supplemented cellulose in *∆bcsA* mutant can lead to biofilm phenotypes that resemble either the co-culture or the WT. Our observations reveal that upon extraneous cellulose addition, the phenotype of the *∆bcsA* mutant biofilm appears somewhat similar to the co-culture but it does not recapitulate the phenotype of WT biofilms (Figure S2). It is important to note that the mature biofilm architecture is missing in the co-culture which implies that other ECM components like extracellular DNA/colanic acid/capsular O antigen is probably differentially expressed in WT relative to mutants [10] or these matrix forming genes might regulate other genes that could possibly contribute to mature biofilm architecture.

Scanning electron microscopy (SEM) was done to visualize the colony biofilm architecture (Fig. 3b) and the results show that the Wild type of colony biofilm produces the ridges and the hollow cavity inside the colony biofilm and the channels can also be viewed. In the mutants, we could not observe any ridges or patterns. *∆csgD* produces a plain colony where the matrix components are absent*, ∆csgA* and *∆bcsA* the periphery of the colony shows folding or wrinkled structures. And in the co-culture *∆csgA: ∆bcsA* the colony shows a more well-defined colony structure like *∆csgA,* but the wrinkled architecture characteristic of WT is still missing, which again reiterates the fact that these matrix contributing genes might also impinge on signalling that results in mature biofilm architecture.

### Antibiotic stress exposure

**Fig. 4:**
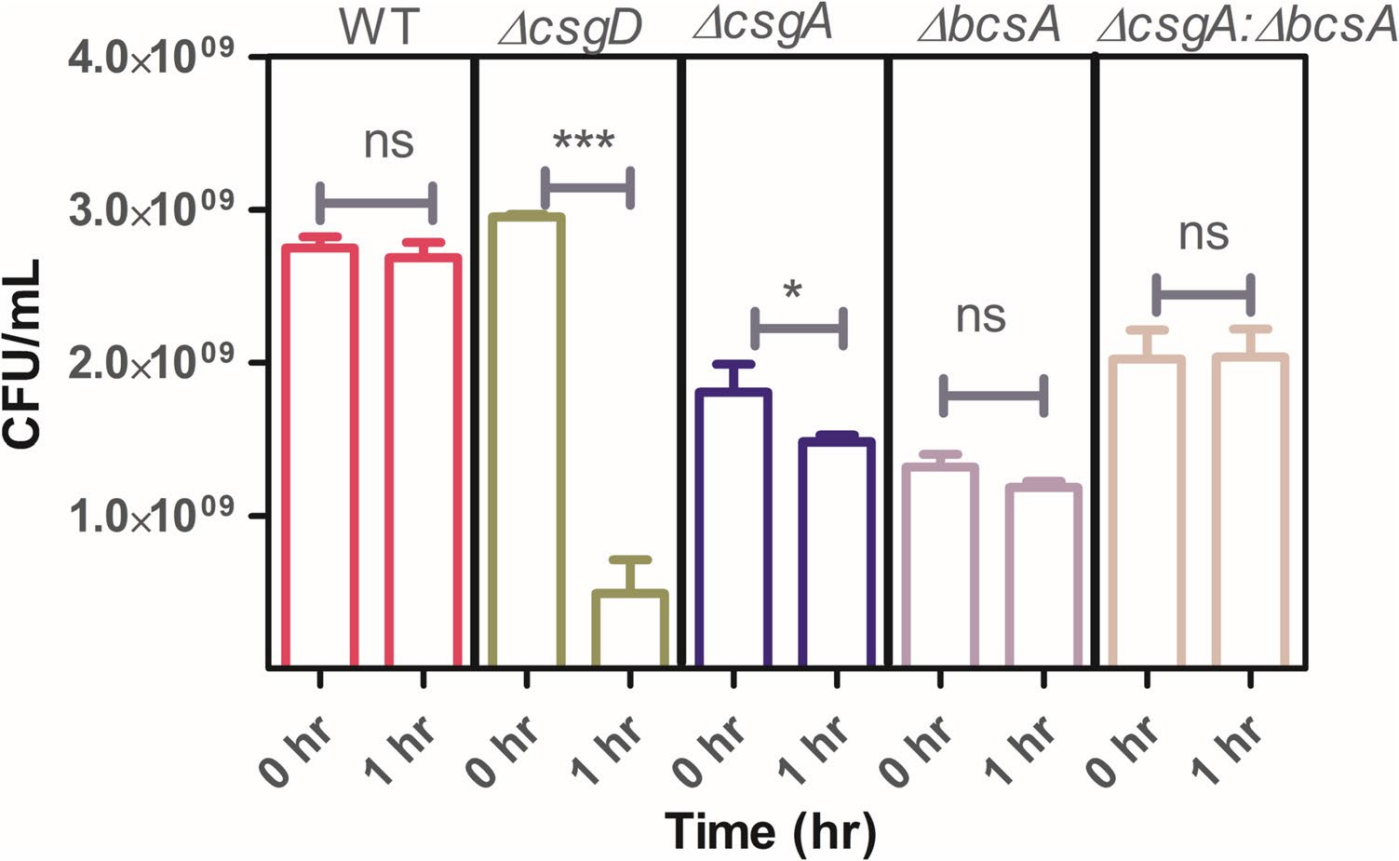
Matrix sharing occurs in co-culture and protects biofilms against antibiotic stress. 0 h is the CFU of cells before antibiotic treatment and 1 h is after antibiotic treatment. In the WT and co-culture, no reduction in CFU was observed due to antibiotic exposure. *n* = 6 biological replicates, Paired *t*-test was performed, 95% CI, *P* value < 0.05

The role of matrix components in protecting biofilm cells from antimicrobial stress was tested by exposing biofilms formed by WT and mutants to 200X MIC of ciprofloxacin. Results show that in the STm—WT exposure to antibiotics did not induce a reduction in CFU/mL indicating that the matrix is protecting the cells from the antibiotic stress. In *∆csgD* there is > 90% reduction in cell counts, which could be attributed to the absence of matrix (Fig. 4). As the cells exposed to antibiotics are vulnerable because of the absence of protection afforded by the matrix, they were killed as reported earlier [7]. With *∆csgA* cellulose affords protection from antibiotics, but the protection is not complete as a 30% decline in cell counts was observed after antibiotic exposure. Similarly, *∆bcsA* curli conferred protection to the cells and antibiotic treated biofilms displayed a 10% reduction relative to untreated *∆bcsA* mutant. Among mutants relative to cellulose mutant, curli mutant confers enhanced protection upon exposure to antimicrobial stress. As the specialists (mutants) lack one of the matrix components, complete protection from antibiotic stress like WT could not be observed. With co-culture of specialists (*∆csgA: ∆bcsA*) where both the matrix components are contributed by individual strains, hardly any decline in cell counts following antibiotic exposure was observed which implies that matrix sharing would have contributed to enhanced antimicrobial resilience similar to WT in the mutant co-cultures.

### Chlorine stress experiment

**Fig. 5:**
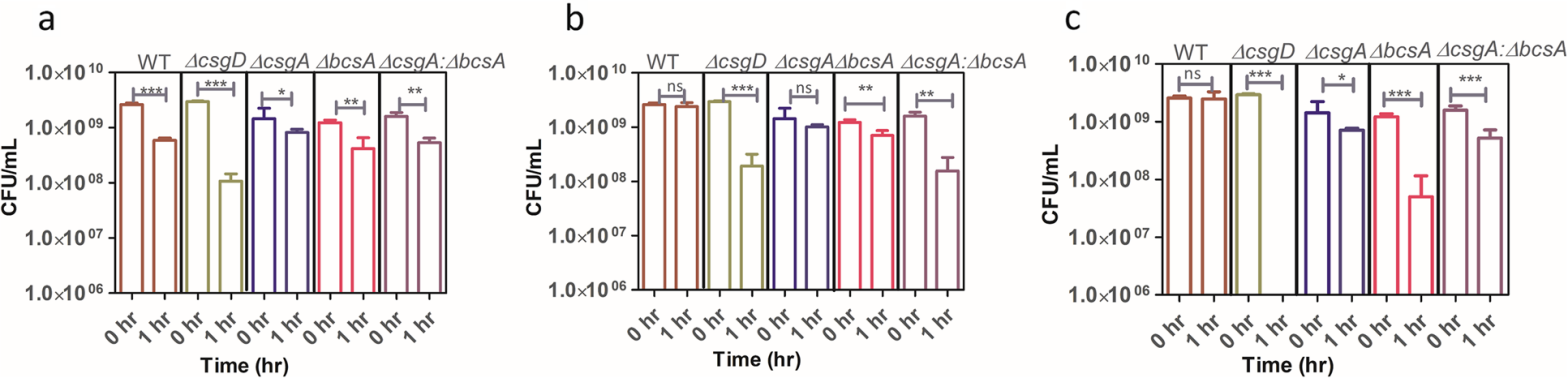
Matrix protects biofilm cells under elevated chloride ion stress. 0 h is the CFU of cells before chlorine treatment and 1 h is the CFU post chlorine treatment. Three different concentrations of sodium hypochlorite (**a** 200, **b** 400, **c** 500 p.p.m) were used. **a** At 200 p.p.m, WT, and co-culture showed a similar pattern of CFU reduction there was less reduction in CFU than *∆csgD* mutant. **b** There was no significant CFU reduction in WT at 400 p.p.m. **c** At 500 p.p.m there was a complete reduction in CFU of the *∆csgD* mutant and in co-culture where both cellulose and curli are produced to complement each other and protect itself from chlorine stress. *n* = 3 biological replicates, Paired *t*-test, 95% CI, *P* value < 0.05

To evaluate protection conferred by the matrix against oxidative stress, colony biofilms of WT and mutants were exposed to different concentrations of Sodium hypochlorite. As expected, the WT protected the cells from oxidative stress due to its matrix producing ability (Fig. 5). In *∆csgD* wherein the matrix is absent, there was a significant decline in cell counts at all concentrations tested, and at the maximal concentration evaluated (500 p.p.m) the *∆csgD* could not survive. In *∆csgA,* cellulose conferred protection to the mutant although a slight decline in cell counts was observed at all concentrations tested, the reduction in cell counts was not drastic post exposure. Whereas in *∆bcsA,* which produced only curli, at lower concentrations cell counts were comparable to *∆csgA* mutant whereas, at 500 p.p.m of Sodium hypochlorite*,* a higher mortality was observed due to oxidative stress relative to *∆csgA* implying that cellulose afforded enhanced protection against oxidative stress than curli. In co-culture *∆csgA: ∆bcsA* the decline in cell counts was similar to what was observed in *∆csgA* (Fig. 5) reiterating the protective role of cellulose against oxidative stress*.* It is unclear if cellulose and curli synergise and form a well interspersed bundle that confers enhanced protection against antibiotic/oxidative stress in the WT relative to the co-culture where both components are present together.

### Myxococcus invasion assay

**Fig. 6:**
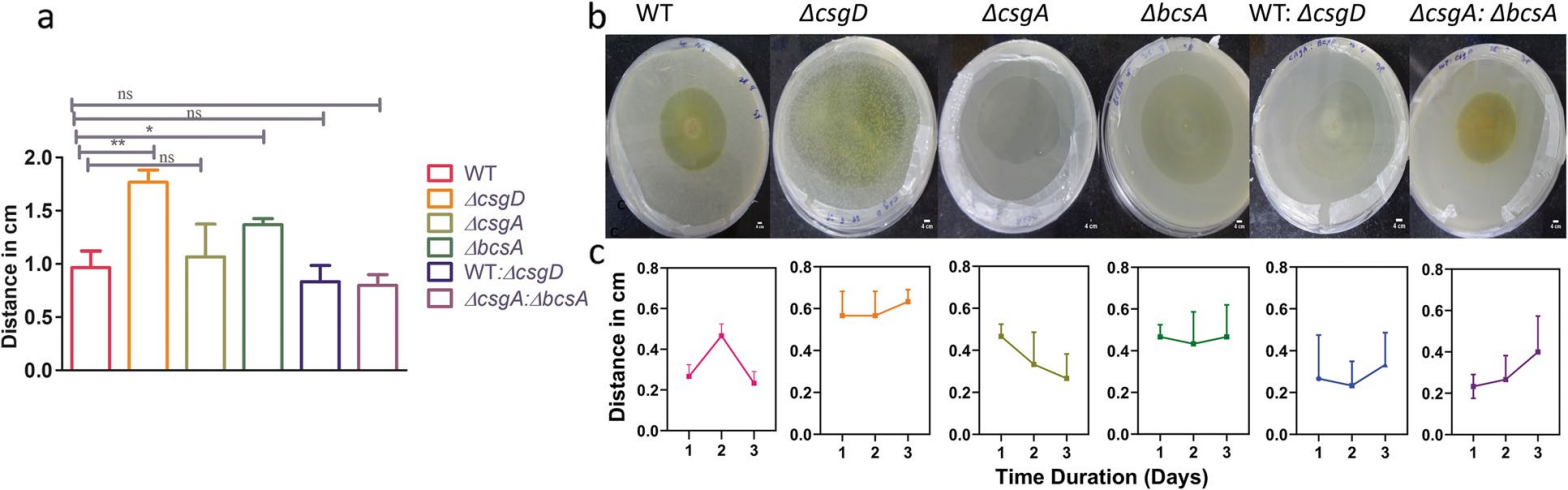
**a** The cumulative distance of predation for the 3 days by the predator. *∆csgD* has no matrix so predation was more. And in other cases, the matrix conferred resilience from predation. In the co-culture, there was much less predation compared to the mutants. One-way ANOVA was performed, 95% CI, *P* < 0.001-***,* P* < 0.01- **, *n* = *3*. **b** Predation assay between *M. xanthus* and STm WT, *∆csgD,* ∆*csgA, ∆bcsA,* WT: ∆*csgD,* ∆*csgA: ∆bcsA* documented on 3rd day. **c** the graph shows the distance of predation for each day in STm WT, *∆csgD,* ∆*csgA, ∆bcsA,* WT: ∆*csgD,* ∆*csgA: ∆bcsA*, Error bar represents SD (*n* = 3)

Swarming assay technique was employed wherein the predator myxococcus spotted in the center predates by swarming and kills the prey. The mutant *∆csgD* lacking the matrix displayed a higher susceptibility towards *Myxococcus* invasion as evidenced by enhanced swarming diameter as shown by the cumulative graph of the 3-day predation assay (Fig. 6a). Co-cultures ∆*csgA: ∆bcsA* and WT: ∆*csgD* afforded significant protection from the predator as evidenced by lower migration rates. It is likely that the sharing of matrix between the mutant co-culture and between WT and matrix deficient mutant affords protection against Myxococcus swarming (predation) (Fig. 6).

### Density of the colony

The cell density of the biofilm was assessed by CFU/mL divided by the diameter of the colony. In general, as the matrix is comprised of polysaccharides/proteins, it is less dense than the cells. ∆*csgD* had no matrix hence it produced cells whose density was the highest. WT had a cell density comparable to *∆csgA*. But the *∆bscA* mutant which produces only curli protein exhibited lower cell density. Co-culture of both mutants produced the lowest cell density of ~ 1 log lower implying that the biofilm biomass is predominantly contributed by the matrix in co-culture (Fig. S3).

### Iron estimation in the matrix of the colony

**Fig. 7:**
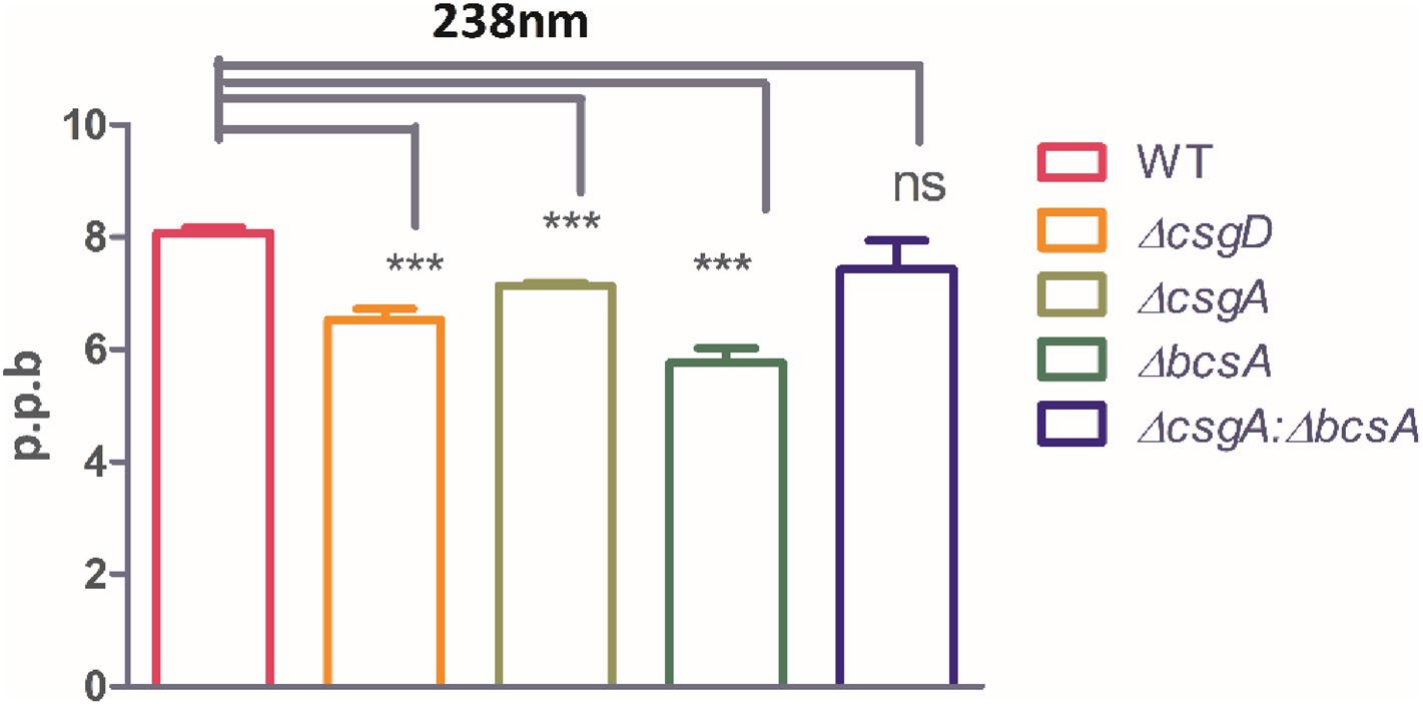
The co-culture of *∆csgA: ∆bcsA* can hold iron equivalent to that of WT. *n* = 3 Unpaired *t*-test, 95% CI, *p* < 0.05 = ***

As iron is critical for microbial metabolism (reduction of oxygen for ATP production, Haem production, Ribotide precursor reduction, etc.,) the role of matrix components in sequestering iron was evaluated. The results reveal that there is more iron sequestered by the WT and co-culture colony biofilm relative to the individual mutants (Fig. 7). In *∆bcsA* the iron acquisition is less, which is similar to matrix deficient mutant *∆csgD*. Relatively *∆csgA* accumulates more iron which implies that cellulose contributes to iron sequestration than curli. Thus, in co-culture *(∆csgA: ∆bcsA*), *∆csgA* by sharing the matrix would have conferred iron chelation ability and balance for *∆bcsA* to succeed as a combined group.

### Stability experiment

To check for the stability of the colony biofilms formed by WT, mutants, and co-cultures, the colony biofilms were subjected to rocker stress at 75 rpm for a period of 3 h. STm- WT even after 3 h was not disrupted and remained intact underscoring the strong influence of the matrix components in conferring structural integrity to the biofilm (Fig. S4). In the case of the mutant ∆csgD, the entire biofilm dispersed within 2 h as they lacked the matrix and could not withstand the stress. With *∆bcsA*, the colony biofilm showed signs of disruption after the second hour, and by 3 h, the entire biofilm was dispersed. In *∆csgA* although the colony biofilm got disrupted by 2 h, it was not completely dispersed even by the 3^rd^ hour. This implies that cellulose confers better structural stability to the colony biofilms. The co-culture *∆csgA: ∆bcsA*, was dispersed by 3^rd^ hour but withstood until the 2 h. So, the co-culture was relatively more stable than *∆csgD* but it did not display the stability observed in wild type biofilms. It is likely that WT has an interspersed matrix whereas co-culture has a layered matrix which differs in its resilience.

### Matrix estimation by congo red depletion assay

The proportion of matrix formed was estimated by Congo red depletion assay wherein enhanced absorbance in supernatant implies reduced binding of the dye with matrix components. Matrix production is seen at the highest in the specialist *∆csgA*, whereas *∆bcsA* the curli producing mutant forms less amount of matrix. In the co-culture *∆csgA: ∆bcsA* poor matrix formation of *∆bcsA* is compensated by *∆csgA*, as the amount of matrix formed is similar to that observed in WT (Fig. S5). Thus, in the co-culture *∆csgA* is sharing its matrix with *∆bcsA* to sustain as a population.

### Cellulose estimation by calcofluor staining method

Calcofluor binds to cellulose and estimation of bound calcofluor by spectrofluorimetry is indirectly proportional to the amount of cellulose formed by different biofilms. The observations reveal that Cellulose is produced in large quantities by *∆csgA* because the mutant is specialized to produce only a single matrix component (cellulose) which is produced in more amounts than the WT. The co-culture *∆csgA: ∆bcsA* produces cellulose comparable to the WT (Fig. S6) wherein the *csgA* mutant contributes exclusively to cellulose production. Sharing of the matrix occurs in co-culture and hence the amount of cellulose formed is comparable between co-culture and WT.

## Discussion

*Salmonella*, a well-studied member of the *Enterobacteriaceae* family featuring a plethora of different serovars has adapted to diverse environmental and lifestyle conditions. The self-produced extracellular matrix that comprises the heterogenous and spatially structured bacterial communities with multiple subpopulations residing in the nanocomposite formed together by the major matrix components, allows the biofilm to act like a tissue that confers resistance against various stressors like antibiotics, environmental stress, and competing host responses. Curli as a protein elicits an immune response in the host and can be easily recognized by the host immune cells. Cellulose counterintuitively masks the curli and prevents it from being recognised by the host, resulting in a successful habitat for coordinated bacterial existence known as biofilms, a community with multiple heterogeneous subpopulations having distinct physiological traits and division of labour which increases pathogen fitness in both host and non-host environments [11, 12].

In higher organisms, there are several examples of honeybees, termite societies, and polar bears where social interactions like competition and cooperation promote the overall fitness of the community [13]. Communal interactions also regulate the mechanism of biofilm development where in a community, the producer protect the non-producer which confers an overall protection to the biofilm community and the biofilm fitness depends on the balance of these interactions. There is physiological differentiation of microbial cells in different biofilm microenvironments which have coordinated behaviours as seen in multicellular organisms [14]. Competition is the majorly observed interaction in the microbial community, but cooperative behaviour is also observed among the kin population [15]. In the present study, we were interested in looking into the role of matrix conferring resistance against the various stress response and whether sharing of the matrix in co-cultures deficient in the production of one of the matrix components confers a fitness advantage to *Salmonella* colony biofilms.

Biofilm exists in nature as air–liquid interface (Pellicle biofilm), solid–liquid (submerged biofilm), and air–solid (colony biofilm). And cells in a biofilm are a highly heterogeneous population. To test for phenotypic heterogeneity and to visualize the production of matrix we spotted these biofilms after serial dilution onto a Congo red media (Fig. S1b). And the expressed phenotypes in each model were recorded (Fig. S1a). In the pellicle biofilm, the bacteria obtain oxygen from the air and nutrients from the liquid. flagella, FimH, cellulose, and curli are important for pellicle formation [16]. And flagella play an important role in the early stages of biofilm formation. mutants lacking these motility genes were defective in pellicle formation in *V. fischeri* [17]. This could be attributed to the fact that motility is important for the cells to reach the air–liquid interface to initiate pellicle formation. Hence, the expression of the SAW phenotype was predominant in pellicle biofilm. In submerged biofilms, where they are attached to a solid substratum, the need for motility is comparatively limited. Only in the initial stages of attachment to the substratum would require motility. So, the expression of RDAR phenotype, which is associated with the expression of both curli, and cellulose offers more advantage where both expression of cellulose and curli occurs simultaneously. In the colony biofilm grown on the solid-air interface, as motility related genes are not expressed a preponderance of RDAR phenotype was observed. STm strain is characterized by the production of RDAR phenotype the multicellular form in the agar media [18].

The Congo red morphotyping Fig. 3a reveals that despite taking up of the Congo red by the co-culture, which implies matrix sharing, the mature biofilm architecture like the wild type was not observed, which implies that complementation by specialists is not adequate to restore intricate biofilm architecture displayed by the wild type. In other words, although co-culture aids in biofilm formation it is unable to complete biofilm maturation observed in wild type. The absence of intricate biofilm architecture displayed by the wild type, reveals that the specialists are unable to recapitulate biofilm architecture displayed by the generalist. Interestingly signs of septum like structures that are well developed in the wild type are observed only in *∆csgA* mutant implying that cellulose predominantly contributes to the mature biofilm architecture. But in a recent study it was observed that the specialist co-culture restored the RDAR phenotype and sharing of the matrix was displayed [19]. Furthermore, This study indicates that there are more factors other than cellulose that give rise to the RDAR phenotype in *E.coli* 0157:H7 [20]. An earlier study showed that the biofilm matrix in non-typhoidal *Salmonella* is predominantly contributed by curli and to a certain extent by colanic acid [10]. Our findings also reveal that though cellulose and curli are important components of the biofilm matrix, there could be other factors like eDNA/Colanic acid/ Capsular O antigen that might contribute to the RDAR morphotype of WT.

The antibiotic prescribed/administered for *Salmonella* related infections is of greater importance because serovars of *Salmonella* have gained resistance to commonly prescribed antibiotics. *Salmonella* infections are usually treated using the fluoroquinolone class of drugs mainly ciprofloxacin, which inhibits bacterial DNA gyrase and Topoisomerase. Studies show that resistance of *Salmonella* serovars to ciprofloxacin is increasing [21, 22]. The bacteria’s virulence and resistance are contributed to a greater extent by the matrix components. Matrix deficient *∆csgD* is reported to be sensitive and WT is tolerant to the antibiotic Ciprofloxacin [19]. The MIC of *S*. Typhimurium 14028 strain for ciprofloxacin is ∼0.02 µg mL^−1^ and the authors of the earlier study have checked for sensitivity at 4 µg mL^−1^ ciprofloxacin in submerged biofilms [7]. To investigate whether matrix production is conferring enhanced protection in colony biofilms against antibiotic, STm WT, *∆csgD, ∆csgA, ∆bcsA, ∆csgA:∆bcsA* colony biofilms were treated with 4 µg mL^−1^ ciprofloxacin (Fig. 4). In the co-culture there is less killing compared to that of the individual mutants.

*∆csgD* mutant is more sensitive to hydrogen peroxide [19]. Cellulose deficient mutants are reported to be more sensitive toward chlorine stress in submerged biofilms [7]. An earlier study checked the survival of *S. enteritidis* in food and water supply chains by exposing the population to a higher concentration of 30 p.p.m chlorine (which is 200 times higher than the free chlorine in municipal water supplies) it was observed that WT had a higher survival upon 30 p.p.m chlorine exposure relative to cellulose deficient mutant which exhibited greater mortality [23]. Interestingly our observations (Fig. 5) show that even though the co-culture is better, it could not fully recapitulate the protective effect conferred by the wild type.

Myxococcus is a generalist bacterium that has a broad host range feeding on bacteria that is in proximity by employing various secondary metabolites and enzymes to lyse the microbial population and obtain its desired nutrients [24]. Matrix components cellulose and curli were shown earlier to provide robust protection to *E.coli* from the predatory bacterium *Myxococcus xanthus* [25]*.* To test whether the matrix is conferring protection against predatory stress in *Salmonella* and to test whether sharing of the matrix that occurs in co-culture affords protection against *Myxococcus*, invasion assay was carried out and results (Fig. 6) revealed that the matrix confers protection as there is far less killing in co-culture biofilms relative to biofilms formed by individual mutants.

Iron is an essential element for the growth of bacteria. A previous study reported that matrix associated iron is used for intracellular electron transfer during oxidative respiration [26]. Our results (Fig. 7) show that both the co-culture and WT acquire more iron relative to iron sequestered by the mutant biofilms. Our observations (Fig. S4) regarding biofilm stability in the face of shear stress created by rocking show that WT colony biofilm is most stable and did not get dispersed whereas co-culture was not fully stable nevertheless, it could not be completely dispersed which implies mild protection against shear stress for a large duration (> 2 h).

Matrix produced in the colony is estimated using congo red method (Fig. S5), where the cellulose specialist produced more cellulose than the WT. Whereas the curli specialist produced lesser curli than the WT but together in co-culture they are equalizing matrix produced by the WT. calcofluor white has been used to identify the mutants defective in producing matrix in many organisms [23]. Paytubi et al., reported that cellulose is not essential for pellicle biofilm formation [27]. On the contrary, Thongsomboon et al., reported that functionally cellulose is the major component of the matrix and plays an important role in biofilm architecture and dictates a framework for biofilm landscape providing structure and protection [11]. Cellulose estimation assay performed in the present study (Fig. S6) showed that *∆csgA* produced a higher amount of cellulose than the WT most likely to compensate for the absence of other matrix components.

In this study, we showed for the first time that matrix aids in the survival of STm WT, *∆csgD*, *∆csgA, ∆bcsA, ∆csgA: ∆bcsA* against antibiotic, chlorine, and *Myxococcus* stress through matrix sharing in the co-culture. Despite the matrix sharing in the co-culture, it is not able to mimic the generalist (WT). An earlier AFM based study on the *Salmonella* biofilm matrix revealed that curli was visible as an extracellular material both on and in between the cells and on the edges of the biofilms as fimbrial curli [28]. Hence, it is likely that curli gets intertwined with cellulose fibrils in WT and affords greater resilience relative to the layered arrangement of curli and cellulose in co-culture, which remains to be discerned using confocal imaging studies as a part of future work.

## Conclusion

Understanding the structure and interactions that occur within a biofilm is critical for studying these highly heterogeneous life forms that switch to a favourable phenotype when necessary [29–31]. Our findings indicate that matrix helps with survival during antibiotic, chlorine, and predatory stress. And possible matrix sharing is taking place, with one counterbalancing the inability of the other during stressful conditions. Studying these various specialists and generalists is important because, during an infection, the bacteria will select a phenotype that will allow it to establish itself. And there will be phenotypic switching to combat stress and conserve metabolic energy. A trade-off can occur among the heterogeneous group in biofilm, where one will produce one metabolic product and complement the non-producer, and they may establish themselves as a community. Cooperation is a potentially advantageous behaviour among the kin population to establish itself during infection and other challenging environments which require further experimentation to unravel it fully.

## Materials and methods

### Bacterial strains and culture conditions

*Salmonella enterica* serovar Typhimurium 14028 strain (STm WT), *∆csgD, ∆csgA, ∆bcsA* [7] were kindly gifted by Prof. Dipshikha Chakravortty, (Indian Institute of Science). For all the experiments strains were grown in LB-NaCl broth overnight at room temperature. 2µL of overnight culture (OD of 0.6 at 595 nm) was spotted onto LB without NaCl agar and incubated for 3 days at room temperature (26- 28 °C) to form colony biofilms [19, 32] which was used for further experiments. All the chemicals and antibiotics used were purchased from HiMedia Labs, India.

### Pellicle biofilm, submerged, and colony biofilm

Pellicle biofilms were grown by adding 2 µL of mid log culture (0.6 OD) to 200 µL LB broth in a 24 well (Tarsons, Kolkata) tissue culture plate wrapped in parafilm for 6 days at 25 °C in static conditions. The pellicle formed from this culture was aspirated into 1 mL Phosphate buffered saline (PBS). The resuspended pellicle was vortexed, serial diluted, and spread plated on Congo Red (CR) media. 3 days post incubation, the morphology of the biofilm colonies was observed and recorded [33].

A similar procedure was adopted for submerged biofilm, wherein rubber policeman was used for retrieving the submerged biofilm, and for colony biofilms, 3-day old colony biofilm was taken which was then added to 1 mL PBS, serially diluted, and plated onto CR media. After 3 days of incubation, the morphology of the colonies was typed and recorded.

### Crystal violet assay

To assess the biofilm formation, crystal violet assay as reported earlier [33, 34] was adopted. Briefly, biofilms were grown on 96 well plate containing 200 µL of LB broth (without NaCl) at 26- 28 °C for 3 days in static conditions. On the 3-day the liquid culture was removed by aspiration, biofilms formed were washed twice with sterile PBS and air dried. 1% crystal violet was added to the tubes and allowed to stain for 15 min, excess/unbound crystal violet was carefully removed from the tubes which were air dried and imaged. 70% ethanol was added to extract crystal violet and the OD of crystal violet corresponding to the biofilm biomass was measured at 595 nm using Tecan Sunrise plate reader [6]. The results were analysed by using *t*-test using GraphPad Prism5 for Windows.

### Morphology changes devoid of matrix

The morphotypes were studied visually on congo red agar plates. 2 µL of STm WT, *∆csgD, ∆csgA, ∆bcsA*, co-culture of *∆csgA: ∆bcsA* (1:1) were spotted on to the LB- NaCl agar media containing 40ug/mL Congo red and 20ug/mL Coomassie blue. The plates were incubated for 3 days. Based on their abilities to produce matrix components, different strains produced varied phenotypes which were photographed and recorded [27].

### Scanning electron microscopy

The 3-day grown colony biofilm was used for SEM preparation and observation following the standard protocol [7] with few modifications. Briefly, the colony biofilm was fixed using 1 mL of warm 25% glutaraldehyde at RT for 10 min. The fixed biofilms were washed and dehydrated using a graded ethanol series of 50% to 100% with each wash step of 10 min. After drying at room temperature, they were left in a vacuum condenser for a day. The samples were sputter coated with gold and imaged using the scanning electron microscope TESCAN VEGA 3, BRNO, CZECH REPUBLIC.

### Antibiotic and chlorine stress exposure

For testing the antimicrobial sensitivity, the colony biofilm was carefully removed, and exposed to Ciprofloxacin at a concentration of 4ug/mL and sodium hypochlorite (200, 400, 500, and 600 p.p.m) for 1 h at 37 °C (Schematic. S1). Untreated colony biofilms in 1 mL PBS were used as the control. Following exposure, the treated and untreated biofilms were mixed well, serially diluted, and plated on LB- NaCl media with respective antibiotic plates, following 24 h of incubation, colony counts were determined to enumerate their CFU. The results were analysed by using paired *t-*test using GraphPad Prism5 for Windows.

### Myxococcus invasion assay

Matrix is known to hinder phagocytosis by macrophages. In a similar manner, to assess the role of matrix against the predatory stress, predation assay was performed using *Myxococcus xanthus*. 100µL of overnight cultures of STm WT, *∆csgD, ∆csgA, ∆bcsA, ∆csgA: ∆bcsA* grown in LB-NaCl was diluted to an OD_600_ of 1 and was spread plated on LB-NaCl agar. 10 µL of *M. xanthus* grown in CTT Liquid (1% Bacto Casitone, 8 mM magnesium sulfate, 10 mM Tris–HCL, 1 mM potassium, pH 7.6) in shaking conditions for 48 h at 30 °C (with a normalized OD_600_ of 1) was spotted in the centre of the plate and the plates were incubated at 30 °C. The distance swarmed by the predator (zone of predation) was measured and recorded for 3 days to see the predatory efficiency of *Myxococcus* on STm WT, mutants, and co-culture [25]. The results were analysed using one-way ANOVA by GraphPad Prism5.

### Density of the colony

The 3-day grown colony biofilms were taken, and the diameter of the colony was measured. The colony was scrapped off carefully, washed with PBS, serial diluted, and plated. The density of the colony was enumerated by dividing the CFUmL^−1^ by the diameter of the colony biofilm. Unpaired *t*-test was performed for result analysis using GraphPad Prism5 for Windows.

### Iron estimation in the matrix of the colony

For estimating the matrix associated iron [26] 3-day old colony biofilm was dispersed in 15 mL PBS, sonicated, and centrifuged at 14,000 rpm for 1 min, the supernatant was collected and filtered using 0.22 µ filter. Fe content in the samples was estimated at 238 nm using ICP-OES Agilent 5110. The statistical test used for analysis is unpaired *t*-test using GraphPad Prism5.

### Stability experiment

To check the role of matrix in stability, STm WT, *∆csgD, ∆csgA, ∆bcsA, ∆csgA: ∆bcsA* were allowed to form colony biofilms for 3 days subsequently. 1.6 mL of PBS was added to the colony biofilms and incubated at the rocker at 75 rpm under shaking conditions for 4 h. The effect of mixing on the stability of the biofilms was observed and photographed at every hour interval.

### Matrix quantification by congo red method

Congo red binding, was used for quantification of matrix production by STm WT, *∆csgD, ∆csgA, ∆bcsA, ∆csgA: ∆bcsA* [32, 35]. Two microliters of overnight grown cultures were spotted in biological triplicates on LB-NaCl Agar and allowed to form biofilms for 3 days at 25 °C. Each colony was scraped from the plate, resuspended in 1 mL PBS + 40 μg/mL Congo red dye, and incubated at 37 °C for 1 h. Samples were centrifuged at 16,873 × g for 2 min, and supernatants were transferred to a clear 96-well plate (Tarsons) for measurement of absorbance at 490 nm using a plate reader (Tecan infinite F50 ELISA Reader). PBS + 40 μg/mL Congo red was measured as the “no matrix” standard. Unpaired t-test was performed using GraphPad Prism5.

### Cellulose quantification by calcofluor staining method

Cellulose production in colony biofilms was quantified by measuring the cellulose bound to cells [27]. STm WT, *∆csgD, ∆csgA, ∆bcsA, ∆csgA: ∆bcsA* were spotted on LB-NaCl Agar supplemented with 2% Calcofluor white stain (Sigma Aldrich), incubated for 3 days at 25 °C. The colony biofilms formed were retrieved, mixed with 1 mL PBS, and centrifuged at 5000 rpm for 10 min to eliminate the unbound cells and Calcofluor. Cells were resuspended in water and transferred to a 96 well plate (Tarsons). Fluorescent measurements corresponding to biofilm bound cellulose (excitation 360 nm, emission 460 nm) were made in Microplate reader (Biotek). The results were analysed using GraphPad Prism5 for Windows.

### Exogenous cellulose supplementation assay

2 µL of *∆bcsA* culture was spotted on LB- NaCl agar plates containing varying concentrations of cellulose (1 mg/mL and 3 mg/mL) [36] which was additionally supplemented with 40ug/mL Congo red and 20ug/mL Coomassie blue for phenotype characterization, the plates were incubated for 3 days to observe and record the morphological changes.

### Supplementary Information

**Additional file 1: Fig S1.** a. Relative proportion of variants in a colony, submerged, and Pellicle biofilms. n=9, 2 way ANOVA was performed, 95% CI, *P* value < 0.05. b. RDAR( Red, dry and rough), SAW( smooth and white), pink dot white, red big colony, hyper swarmer, morphotypes expressed when plated on congo red media. **Fig S2.** Exogeneous Cellulose Supplementation assay a. *∆bcsA* + 0mg/mL, b. *∆bcsA* grown in 1mg/mL cellulose, c. *∆bcsA* grown in 3 mg/mL cellulose, d. representative image of the co-culture *∆csgA*: *∆bcsA* grown in the absence of cellulose, e. representative image of WT grown in the absence of cellulose. **Fig S3.** The density of the colony is found to be more in *ΔcsgD* followed by WT, *ΔcsgA*, *ΔbcsA*, *ΔcsgA*: *ΔbcsA*. The coculture is found to be the least dense. Unpaired *t*-test, *p* <0.0001, *n*=6. **Fig S4.** Stability of Biofilm Colonies. The colonies are tested for their stability for a time of 3 hours. WT survives and floats like a lotus leaf followed by *ΔcsgA* which did not dissolve in the liquid followed by *ΔcsgA*: *ΔbcsA*, *ΔbcsA*, *ΔcsgD*. **Fig S5.** Matrix production was estimated using Congo red method. *ΔcsgA* has more matrix production than the WT. The coculture *ΔcsgA*: *ΔbcsA* has relatively higher matrix production than the *ΔbcsA* mutant. Unpaired *t*-test, *n*= 3, 95% CI, *p* < 0.0001. **Fig S6.** Cellulose production was estimated using the calcofluor staining method where calcofluor selectively binds to the cellulose. *ΔcsgA* produces a higher amount of cellulose than the WT and the coculture *ΔcsgA*: *ΔbcsA* is producing a relatively equal amount of cellulose produced by the WT. *n* = 3, Unpaired *t*-test, Mean + SD, *p* < 0.01= ***.

## Data Availability

The data presented in this study are available from the corresponding author upon request.

## Abbreviations

STm- WT: *Salmonella enterica* Serovar Typhimurium- Wild type
LB: Luria Bertani
CR: Congo red
RT: Room temperature
PBS: Phosphate buffered saline
SEM: Scanning electron microscopy
RDAR: Red dry and rough
SAW: Smooth and white
PDAR: Pink dry and rough
BDAR: Brown dry and rough
