# Supplementary material for: “Sharing the matrix” – a cooperative strategy for survival in Salmonella enterica serovar Typhimurium
Source: BMC Microbiol. 2023 Aug 23;23:230. doi: 10.1186/s12866-023-02972-0 (PMC10463773; doi:10.1186/s12866-023-02972-0)
Supplement: Supplementary file 1 — Additional file 1: Fig S1. a. Relative proportion of variants in a colony, submerged, and Pellicle biofilms. n=9, 2 way ANOVA was performed, 95% CI, P value < 0.05. b. RDAR( Red, dry and rough), SAW( smooth and white), pink dot white, red big colony, hyper swarmer, morphotypes expressed when plated on congo red media. Fig S2. Exogeneous Cellulose Supplementation assay a. ∆bcsA + 0mg/mL, b. ∆bcsA grown in 1mg/mL cellulose, c. ∆bcsA grown in 3 mg/mL cellulose, d. representative image of the co-culture ∆csgA: ∆bcsA grown in the absence of cellulose, e. representative image of WT grown in the absence of cellulose. Fig S3. The density of the colony is found to be more in ΔcsgD followed by WT, ΔcsgA, ΔbcsA, ΔcsgA: ΔbcsA. The coculture is found to be the least dense. Unpaired t-test, p <0.0001, n=6. Fig S4. Stability of Biofilm Colonies. The colonies are tested for their stability for a time of 3 hours. WT survives and floats like a lotus leaf followed by ΔcsgA which did not dissolve in the liquid followed by ΔcsgA: ΔbcsA, ΔbcsA, ΔcsgD. Fig S5. Matrix production was estimated using Congo red method. ΔcsgA has more matrix production than the WT. The coculture ΔcsgA: ΔbcsA has relatively higher matrix production than the ΔbcsA mutant. Unpaired t-test, n= 3, 95% CI, p < 0.0001. Fig S6. Cellulose production was estimated using the calcofluor staining method where calcofluor selectively binds to the cellulose. ΔcsgA produces a higher amount of cellulose than the WT and the coculture ΔcsgA: ΔbcsA is producing a relatively equal amount of cellulose produced by the WT. n = 3, Unpaired t-test, Mean + SD, p < 0.01= ***. [file 12866_2023_2972_MOESM1_ESM.pdf]

SUPPLEMENTARY FILES:

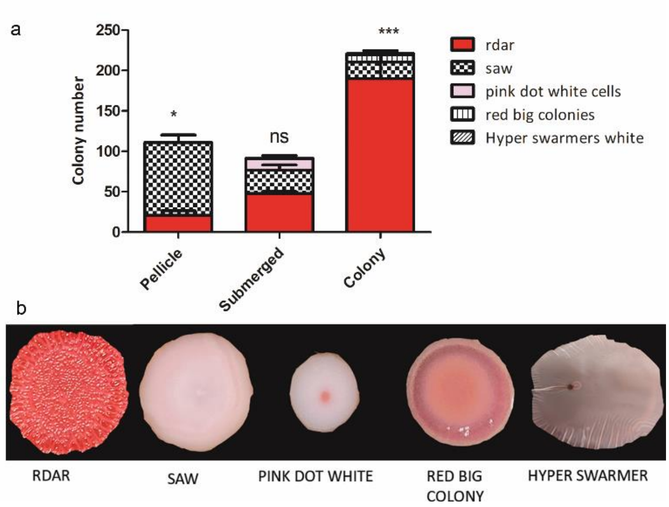

Fig S1. a. Relative proportion of variants in a colony, submerged, and Pellicle biofilms. n=9, 2 way ANOVA was performed, 95% CI, *P* value< 0.05. b. RDAR(Red, dry and rough), SAW(smooth and white), pink dot white, red big colony, hyper swarmer, morphotypes expressed when plated on congo red media.

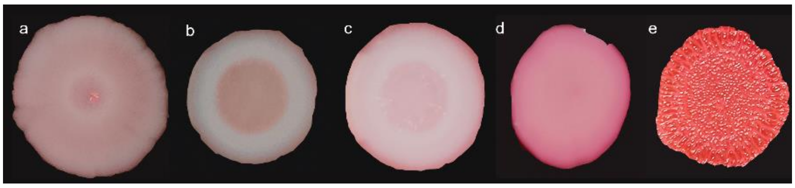

Fig S2. Exogeneous Cellulose Supplementation Assay a. *ΔbcsA* + 0mg/mL, b. *ΔbcsA* grown in 1mg/mL cellulose, c. *ΔbcsA* grown in 3 mg/mL cellulose, d. representative image of the co-culture *ΔcsgA: ΔbcsA* grown in the absence of cellulose, e. representative image of WT grown in the absence of cellulose.

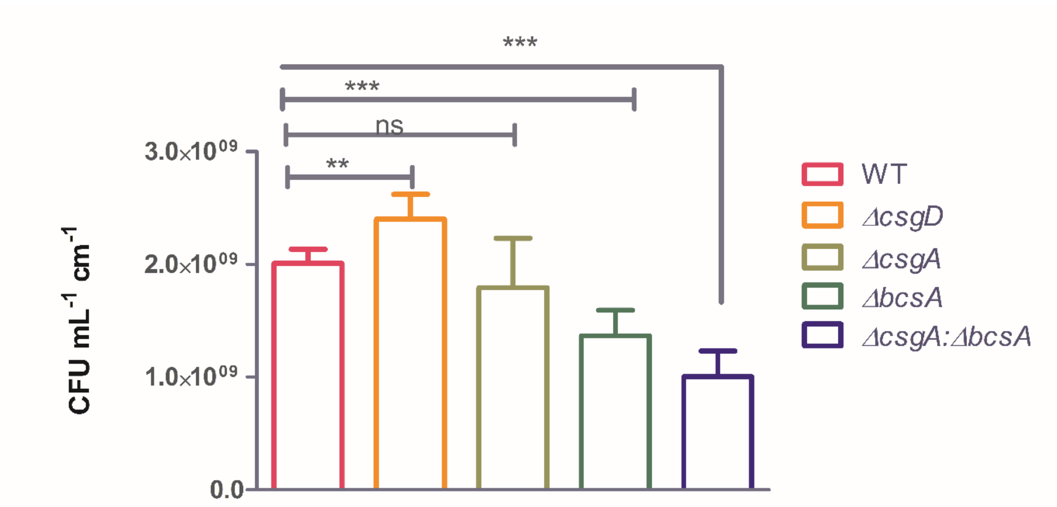

Fig S3: The density of the colony is found to be more in *ΔcsgD* followed by WT, *ΔcsgA, ΔbcsA, ΔcsgA: ΔbcsA*. The coculture is found to be the least dense. Unpaired *t*-test, $p<0.0001$, $n=6$.

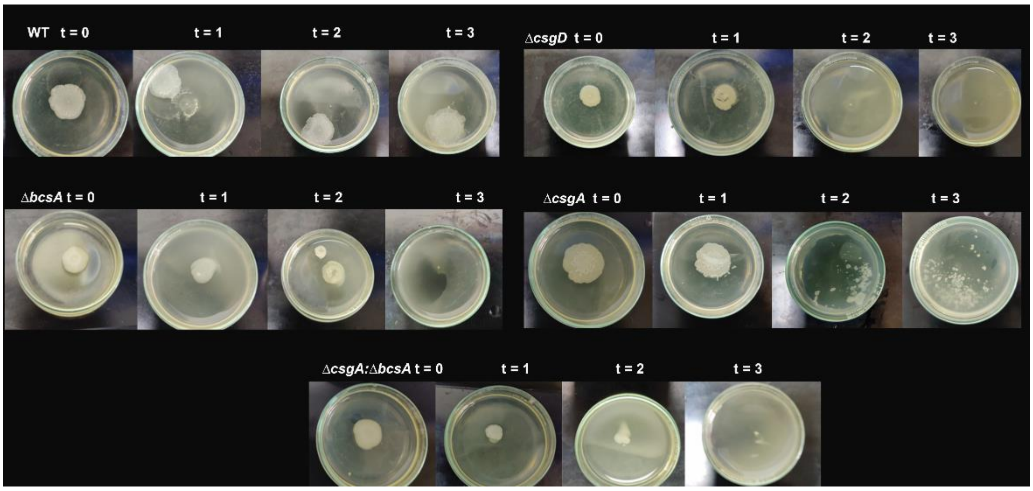

Fig S4: Stability of Biofilm Colonies. The colonies are tested for their stability for a time of 3 hours. WT survives and floats like a lotus leaf followed by Δ*csgA* which did not-dissolve in the liquid followed by *ΔcsgA: ΔbcsA, ΔbcsA, ΔcsgD.*

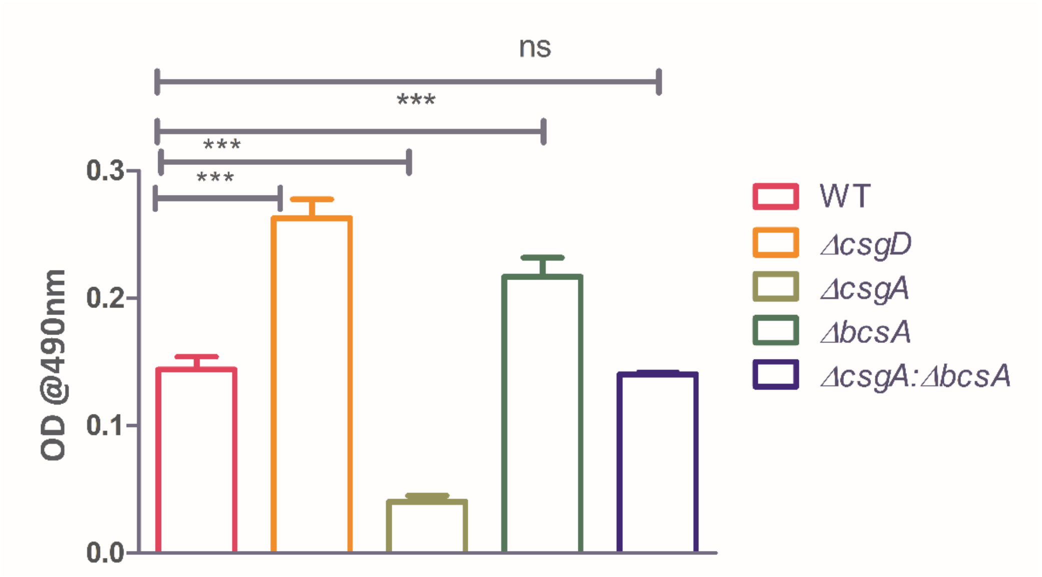

Fig S5: Matrix production was estimated using Congo red method. *ΔcsgA* has more matrix production than the WT. The coculture *ΔcsgA: ΔbcsA* has relatively higher matrix production than the *ΔbcsA* mutant. Unpaired *t*-test, n= 3, 95% CI, $p < 0.0001$

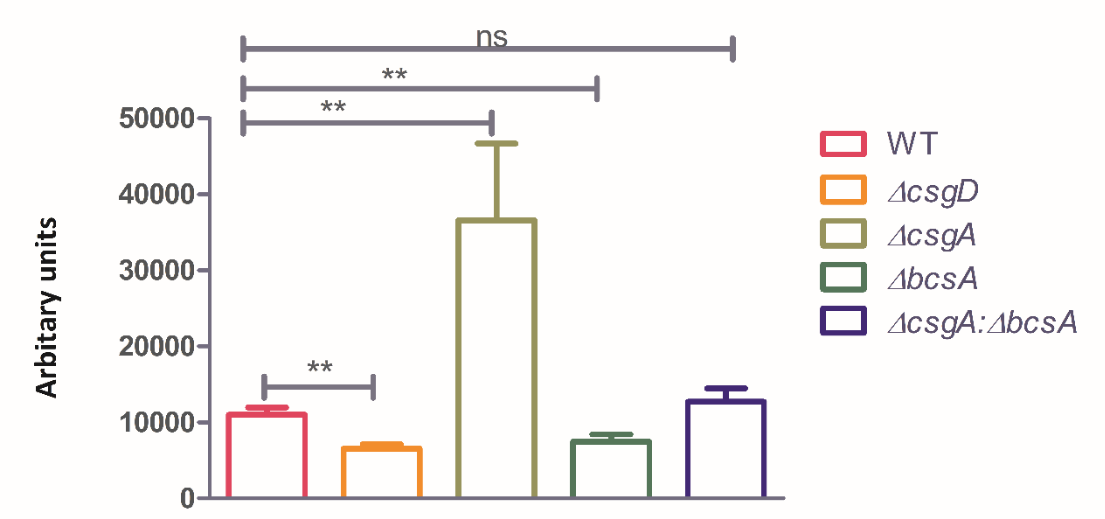

Fig S6: Cellulose production was estimated using the calcofluor staining method where calcofluor selectively binds to the cellulose. *ΔcsgA* produces a higher amount of cellulose than the WT. and the coculture *ΔcsgA: ΔbcsA* produces a relatively equal amount of cellulose produced by the WT. n = 3, Unpaired *t*-test, Mean $\pm$ SD, $p < 0.01$= ***
